# Serum erythropoietin level predicts the prognosis of chronic heart failure with or without anemia

**DOI:** 10.3892/etm.2013.1307

**Published:** 2013-09-18

**Authors:** LING GUO, AI-HONG WANG, YONG-LE SUN, LIN LV, CHONG-EN XU

**Affiliations:** 1 Departments of Cardiology, Provincial Hospital Affiliated to Shandong University, Jinan, Shandong 250021, P.R China; 2 Cardiac Surgery, Provincial Hospital Affiliated to Shandong University, Jinan, Shandong 250021, P.R China

**Keywords:** heart failure, erythropoietin, N-terminal pro-B-type natriuretic peptide, high sensitivity C-reactive protein, prognosis

## Abstract

The aim of this study was to explore the correlation of erythropoietin (EPO) with N-terminal pro-B-type natriuretic peptide (NT-proBNP) and high sensitivity C-reactive protein (hs-CRP) in patients with chronic heart failure (CHF) or CHF complicated with anemia, in addition to its correlation with the prognosis of the patient. A total of 217 CHF patients were enrolled in this study. The patients were graded according to the cardiac function criteria of the New York Heart Association (NYHA). The serum EPO, NT-proBNP and hs-CRP levels of the patients were determined. The patients were followed up for ≥24 months. The EPO expression level in patients with NYHA II–IV CHF was significantly higher compared with that in the control group (P<0.05). EPO expression increased with the aggravation of CHF, exhibiting significant differences amongst the various NYHA graded groups (P<0.05). The EPO expression level increased significantly with an increase in NHA grade in addition to the severity of the anemia in the patients with CHF complicated by anemia (P<0.05). In the patients who succumbed (mortality group), the expression level of EPO was significantly higher and the hemoglobin level was significantly lower compared with those of the survival group (P<0.05). The EPO expression levels were elevated in CHF patients and patients with CHF and anemia. The level of expression correlated positively with the severity of CHF as well as that of anemia. Serum EPO measurements were successful in predicting the mortality and re-hospitalization rates of CHF patients at the end point, within two years of follow-up.

## Introduction

The treatment of chronic heart failure (CHF) is currently an area of interest in the field of cardiovascular disease. Numerous factors such as neurohormonal activation, inflammation and oxidative stress may lead to a poor prognosis of CHF and a one-year mortality rate as high as 30–40% (1,2). Although drug treatments for CHF have improved markedly over the last decade, ~20% of patients require regular in-hospital treatments every year due to recurrent attacks, a consequence of which is that the disease develops into a refractory condition and is ultimately fatal. Currently, a high number of patients with CHF are awaiting medical treatment. For these patients, finding correctable curative factors and then curing or relieving related symptoms is vital; among these factors is anemia (3,4). Anemia has an incidence rate of between 40 and 50% in patients with CHF, which increases with the increased severity of CHF (5–7). The incidence rate may be as high as 80% in patients with heart function NYHA IV. Anemia is an independent risk factor amongst the various factors that induce CHF-related mortalities and has the potential to double the mortality rate (8,9). Anemia-associated histanoxia causes vasodilatation, which activates the sympathetic nervous system, leading to peripheral vasoconstriction. Kidney vasoconstriction subsequently activates the renin-angiotensin-aldosterone system which further aggravates kidney and peripheral vasoconstriction due to an increase in angiotensin II; in the long-term this is harmful for the heart (10,11). However, the significance of anemia in the treatment and deterioration of CHF has been neglected. Furthermore, anemia aggravates the symptoms of CHF and makes its treatment increasingly difficult. Anemia in patients with CHF is primarily caused by a deficiency of erythropoietin (EPO) or the decreased sensitivity of the spinal cord to EPO, and the application of EPO in the treatment of CHF complicated by anemia may have a marked curative effect (12–15).

Neurohormonal and inflammatory activation may trigger CHF and scholars have long sought serological markers capable of reflecting the effectiveness of CHF treatment and predicting its long-term prognosis (16–18). The severity of anemia positively correlates with that of CHF; a higher grade of CHF, according to the cardiac function standard of the New York Heart Association (NYHA), indicates lower hemoglobin (Hb) levels, which indicates a poorer prognosis (5,19,20). EPO levels in the blood may predict the mortality and hospitalization risk for CHF patients in the years following treatment.

EPO, which is produced by the kidneys, promotes the proliferation and differentiation of erythroid progenitor cells in the spinal cord. Recently, with a deepened understanding of its action mechanisms and its potential actions as a multifunctional cytokine, EPO has attracted an increasing amount of attention. EPO has anti-apoptotic and anti-oxidative properties and promotes the activation of endothelial precursor cells for angiogenesis. Due to its multiple properties, EPO has been assumed to be a stress hormone; EPO is highly expressed under aggravated CHF conditions (21–23). A close correlation between EPO and CHF has previously been demonstrated. However, since the overexpression of EPO may also be induced by other stimuli related to tissue hypoperfusion, including oxidative stress and the subsequent production of oxygen radicals, the predictive value of EPO for the prognosis of CHF requires verification.

Therefore, in the current study, the EPO levels of patients with CHF were determined in order to explore the correlation between EPO expression and the severity of CHF and CHF complicated with anemia. The results were analyzed further using known neurohormonal and inflammatory activation markers used for CHF prognostic prediction. The findings of this study may provide theoretical references for the early intervention and prognostic prediction of CHF.

## Subjects and methods

### Subjects

A total of 217 CHF patients who received treatment at Shandong Provincial Hospital (Jinan, China) between January 2007 and December 2010 were recruited for this study. All patients met the Framingham diagnostic criteria of CHF (3) and were graded II–IV, according to the cardiac function standard of the NYHA. In total, 69 patients were diagnosed with accompanied anemia (Hb concentration, <110 g/l).

The study was conducted in accordance with the Declaration of Helsinki and approved by the Ethics Committee of Shandong Provincial Hospital. Written informed consent was obtained from all participants.

### Exclusion criteria

Patients who met any of the following criteria were excluded from the study: age, <18 years; currently pregnant; diagnosed with a malignant tumor; presented with a systemic infection; exhibited gastrointestinal bleeding; and had undergone a cerebral vascular accident, major surgery, or recombinant EPO treatment within the previous three months.

### Medical history

The patients received clinical examinations and were subsequently NYHA-graded. All patients were consulted further, every 1–3 months or dependent on the pathogenic condition. The control group comprised 50 non-CHF patients who were admitted to hospital during the same period exhibiting symptoms of palpitations and chest pain. No significant differences in age or gender were observed between the groups.

### Follow-up

The end point of this study was the time to mortality due to any cause or re-hospitalization due to CHF. All patients were followed-up for 24 months.

### EPO determination

Fasting ulnar venous blood (3 ml) was extracted in the morning for all participants and subsequently centrifuged at 730 × g for 10 min. The serum was separated and stored at −20°C. Serum EPO levels were determined using an radioimmunoassay.

### Hb, N-terminal pro-B-type natriuretic peptide (NT-proBNP) and high sensitivity C-reactive protein (hs-CRP) analysis

Hb content was determined using a Coulter^®^ full-automatic hematology analyzer (Beckman-Coulter, Miami, FL, USA) using the cyan-methemoglobin method. The serum level of NT-proBNP was determined by ELISA. hs-CRP levels were determined using a latex particle-enhanced turbidometric immunoassay.

### Statistical analysis

Data were analyzed using SPSS 10.0 software (SPSS, Inc., Chicago, IL, USA). Enumeration data were presented as the mean ± SE. The Student’s t-test was performed for pairwise comparisons and one-factor ANOVA was performed in order to compare the EPO levels among groups with different NYHA grades. P<0.05 was considered to indicate a statistically significant difference.

## Results

### General patient data

The mean age of the 217 CHF patients was 69.7±10.4 years. According to the NYHA criteria, 74 patients (34.1%) were grade II, 96 (44.2%) were grade III, and 47 were grade IV (21.7%). Of all the patients, 69 (31.8%) had CHF accompanied by anemia. The clinical features and medications of the patients are summarized in Table I, and comparisons of the EPO, NT-proBNP and hs-CRP levels among different NYHA graded groups are summarized in Table II.

### Correlation of EPO with Hb and NYHA grading in CHF patients with anemia

In total, 69 of the 217 patients were diagnosed with CHF accompanied by anemia, which corresponded to an incidence of 31.8%. The EPO level increased with an increase in the NYHA grade in addition to the severity of anemia, demonstrating significant differences among the various groups (P<0.05). The results are summarized in Table III.

### Comparisons between the EPO and Hb levels of the survival and mortality groups

All patients were followed-up through phone calls, clinical visits or hospitalized medical records. During the follow-up period, 34 of the 217 patients succumbed, and 183 survived with an average follow-up time of 3.5±1.3 years. The EPO expression level of the mortality group was significantly higher, compared with that of the survival group, whereas the Hb level was significantly lower, (P<0.05). The results are summarized in Table IV.

### Correlation of EPO, NT-proBNP and hs-CRP with patient prognosis

Of the 217 patients, 34 (15.6%) succumbed, 67 (30.9%) were re-hospitalized due to CHF and 42 (19.4%) were hospitalized due to severe CHF. The mean levels of EPO, NT-proBNP, and hs -CRP at the various end points are summarized in Tables V and VI. The results indicate that the EPO level is relatively successful in predicting the mortality and re-hospitalization rates of CHF patients at end points within two years of follow-up (P<0.01), and NT-proBNP levels may also be used as a successful predictor.

## Discussion

In this study, the serum EPO levels of patients with CHF of various NYHA grades were determined, and compared with those of non-CHF patients who were recruited as controls. The results show that the EPO levels in patients with NYHA II-IV CHF are significantly higher than those in observed in non-CHF patients (P<0.05). In addition, EPO levels significantly increase with an increase in NYHA grade (P<0.05). Furthermore, this study demonstrates that with an increase in NYHA grade, Hb levels decrease significantly. These results demonstrate that EPO is important in the development of CHF. As anoxia is a vital regulatory factor of EPO, patients with CHF presumably suffer from tissue hypoperfusion, which causes internal environmental hypoxia and subsequently leads to the overexpression of EPO. Alternatively, tissue hypoperfusion may lead to other types of stimulation that upregulate the expression of EPO, such as oxidative stress leading to the production of oxygen radicals (24,25).

Epidemiological data have indicated that being female, increased age, kidney insufficiency, low body weight and severe cardiac dysfunction are high risk factors for CHF complicated by anemia (26). The incidence of anemia increases with an increase in the severity of CHF, and may achieve an incidence of 79.1% in patients with NYHA IV CHF. In this study, 69 patients (31.8%) presented with CHF complicated by anemia, and their EPO levels increased significantly with the increased severity of CHF (P<0.05). This finding indicates that EPO is expressed at elevated levels in patients with CHF complicated by anemia and correlates positively with the severity of CHF. Serum EPO is an efficient predictor of the mortality and re-hospitalization rates of CHF patients. The increased expression of serum EPO correlates with a poor CHF prognosis. Furthermore, EPO expression is independent of Hb and other markers that have been confirmed to indicate the severity of CHF.

In addition, this study reveals that serum EPO expression levels correlate with the severity of CHF predicted using NYHA grading. The two years of follow-up in this study further demonstrates that NT-ProBNP expression is an independent predictor of the mortality and re-hospitalization rates of patients with CHF. Furthermore, this study demonstrates that serum EPO expression may serve as a predictor in this respect, whereas hs-CRP levels do not successfully predict the mortality rate.

In conclusion, EPO expression is increased in patients with CHF and in those with CHF complicated by anemia, and the increase correlates with the severity of CHF. Thus, EPO levels may successfully predict the prognosis of CHF, and serum EPO expression performs an important role in the progression of CHF. Furthermore, EPO, NT-ProBNP and hs -CRP correlate with one another. These findings indicate that neurohormonal activation and inflammation are clinical responses to late CHF. Therefore, the determination of serum EPO expression is clinically significant for the prediction of the development, outcome and prognosis of CHF.

## Figures and Tables

**Table I.** Clinical features and medications of 217 CHF patients.

A. Clinical features	
Features	Patient number

Age (years; mean ± SE)	69.7±10.4
Males	145 (67.0%)
Left ventricular ejection fraction	
(mean ± SD)	36±12.6
Hyperlipidemia	118 (54.3%)
Anemia	69 (31.8%)
Kidney failure	85 (39.2%)
Smoking	87 (40.1%)
Hypertension	131 (60.4%)
Diabetes	74 (34.1%)
Ischemic heart disease	160 (73.7%)
Chronic atrial fibrillation	66 (30.4%)
Transient ischemic attack/cerebrovascular accident	25 (11.5%)
Percutaneous transluminal coronary angioplasty or coronary artery bypass surgery	51 (23.5%)

B. Medication	
Drugs	Patient number

ACE inhibitors/ARBs	156 (71.9%)
β-blockers	135 (62.2%)
Spironolactone	83 (38.2%)
Diuretics	151 (69.6%)
Digoxin	55 (25.3%)

CHF, chronic heart failure; ACE, angiotensin-converting enzyme; ARBs, angiotensin receptor blockers.

**Table II.** NYHA grading and EPO, NT-proBNP and hs-CRP levels upon hospital admission.

NYHA grading	Cases	EPO (mU/ml)	NT-proBNP (pg/ml)	hs-CRP (mg/l)
Control	50	2.36±0.73	149.27±68.31	1.60±0.41
NYHA II	74	14.10±1.63	512.34±107.31	2.40±0.32
NYHA III	96	26.70±2.94	2217.00±294.35	3.60±0.54
NYHA IV	47	35.90±5.81	5951.00±424.62	4.50±0.48
P-value		<0.01	<0.01	<0.05

NYHA, New York Heart Association; EPO, erythropoietin; NT-proBNP, N-terminal pro-B-type natriuretic peptide; hs-CRP, high sensitivity C-reactive protein.

**Table III.** Correlations of EPO and Hb with NYHA grading in patients with CHF complicated with anemia.

Groups	Cases	EPO (mU/ml)	Hb (g/dl)
NYHA II	9	14.65±4.53^a^	13.16±2.19^a^
NYHA III	28	27.94±8.46^a^	11.68±2.03^a^
NYHA IV	32	38.47±11.83^a^	10.27±1.94^a^
P-value		<0.05	<0.05

Values are provided as the mean ± SD.

a P<0.05, compared with the next higher NYHA grade. NYHA, New York Heart Association; EPO, erythropoietin; CHF, chronic heart failure; Hb, hemoglobin.

**Table IV.** Comparisons between the EPO and Hb levels of the survival and mortality groups.

Groups	Cases	EPO (mU/ml)	Hb (g/dl)
Survival	183	17.48±5.29	12.64±2.26
Mortality	34	36.74±9.77^a^	10.56±2.03^a^

a P<0.05, compared with the survival group. EPO, erythropoietin; Hb, hemoglobin.

**Table V.** Comparisons between the EPO, NT-proBNP and hs-CRP levels of surviving patients and patients who succumbed to CHF.

Event end point	Cases	EPO (mU/ml)	NT-proBNP (pg/ml)	hs-CRP (mg/l)
Mortality	34	36.74±9.77	6451.00±467.82	5.10±0.44
Survival	183	17.48±5.29	2493.00±329.43	4.20±0.75
P-value		<0.001	<0.001	0.59

EPO, erythropoietin; NT-proBNP, N-terminal pro-B-type natriuretic peptide; hs-CRP, high sensitivity C-reactive protein; CHF, chronic heart failure.

**Table VI.** Comparisons between the EPO, NT-proBNP and hs-CRP levels of re-hospitalized and re-hospitalization-free patients

Event end point	Cases	EPO (mU/ml)	NT-proBNP (pg/ml)	hs-CRP (mg/l)
Mortality or re-hospitalization	143	29.86±9.40	3577.00±415.33	4.30±0.73
Survival and hospitalization-free	74	12.60±5.62	1147.00±307.46	3.70±0.61
P-value		<0.01	<0.01	0.34

EPO, erythropoietin; NT-proBNP, N-terminal pro-B-type natriuretic peptide; hs-CRP, high sensitivity C-reactive protein.
